# Back to the Future With Co-Stimulation Blockade

**DOI:** 10.3389/ti.2023.11752

**Published:** 2023-07-24

**Authors:** Stuart M. Flechner, Klemens Budde

**Affiliations:** ^1^ Cleveland Clinic Lerner College of Medicine, Cleveland, OH, United States; ^2^ Department of Nephrology, Charité Universitätsmedizin Berlin, Berlin, Germany

**Keywords:** immunosuppression, kidney transplant, belatacept, abatacept, co-stimulation blockade

The COVID pandemic that first gripped the world in 2020 caused social and economic dislocations; including healthcare in general, and clinical transplantation in particular [1]. Many transplant centers tried to reduce hospital admissions and direct contact between patients and providers, and there was widespread expansion of telehealth and remote medical services. During these times kidney transplant recipients receiving monthly intravenous injections of the co-stimulation blocker Belatacept were identified as high risk for hospital-acquired COVID infection and as needing an alternative to hospital-based i.v. drug injections. While some centers converted patients back to oral immunosuppressive protocols [2] others initiated a specific infection control protocol [3]. In the current issue of Transplantation International two French transplant centers [4] describe an alternative approach and converted 176 patients from maintenance 5 mg i.v. monthly in-center Belatacept to weekly 125 mg subcutaneous Abatacept injections at home. The reason for the drug switch was to reduce the need for patients to travel to the transplant center during the early chaotic period of the COVID pandemic. It is important to note that patients were previously converted to Belatacept because of calcineurin inhibitor (CNI) toxicity (mean eGFR 38 mL/min), and were also given mycophenolate mofetil and steroids. The authors postulated that the alternative CD80/86 co-stimulation blocker Abatacept, administered subcutaneously, could substitute for i.v. Belatacept and provide equivalent immunosuppression using the approved dose for rheumatoid arthritis [5]. After 3 months patients were reconverted to Belatacept, when in-home infusions of Belatacept were again authorized in France. During this short 3-month observation period a low frequency (1%–2%) of changes in graft function, rejection, viral infections, and adverse events were recorded. Injection site reactions were uncommon and not severe. Seven patients (4%) experienced COVID-19 while treated with Abatacept, two developed severe symptoms but all recovered. Importantly, the patients were well informed and felt safe after conversion to Abatacept. In 61% of patients home care nurses did the injections, and approximately half of the patients found Abatacept injections less restrictive because of independence and no hospital attendance. Interestingly, 49% of patients would continue with Belatacept if they had the choice, compared to only 38% with Abatacept. Patients who preferred Belatacept reported that they liked hospital-based reassurance of their status, and disliked weekly injections, nurse dependency, and the risk of forgetting. In summary, this large well-described cohort demonstrates the feasibility, safety, and efficacy of once-weekly subcutaneous injection of Abatacept in kidney transplant recipients previously treated with Belatacept. Although some patients still favored the less frequent but more intrusive hospital-based i.v. method of drug delivery.

Belatacept, a fusion protein of the Fc fragment of a human IgG1 linked to the extracellular domain of CTLA-4 [6] was engineered to overcome slightly weaker binding avidity to CD80/86 of the progenitor molecule Abatacept, which differs just by 2 amino acids. These changes increased *in vitro* T-cell inhibitory activity and demonstrated superior protection from allograft rejection in pre-clinical models but were limited to the i.v. formulation of Belatacept. Extensive clinical trials and follow-ups have confirmed the efficacy of Belatacept for kidney transplantation [6–8], and the drug is often employed as a substitute or conversion agent to overcome intolerance to CNIs. Common reasons for switching include nephrotoxicity, thrombotic microangiopathy, posterior reversible encephalopathy syndrome, reducing cardiovascular risk factors, etc. However, widespread use has been limited by the need for monthly i.v. infusions, production shortages, and concerns of more acute rejection in higher immunological risk patients [9]. Subcutaneous Abatacept fell off the transplant radar but did find a home to treat autoimmune disorders such as rheumatoid arthritis, psoriasis, autoimmune cytopenia, and others [5, 10]. Over the last decade, several small case series and case reports described the use of Abatacept in kidney transplantation for narrow indications such as recurrent focal segmental glomerulosclerosis (FSGS) [10–13], due to unavailability of Belatacept [14] or the lack of venous access [15]. While results on the treatment of recurrent FSGS with Abatacept are conflicting, all the reports confirm overall safety and effective prevention of rejection with Abatacept, comparable to Belatacept. The largest series until now included 9 rescue kidney recipients switched from a CNI to Abatacept when Belatacept was unavailable. This resulted in stable graft function for a median of 82 months (14). Most (8/9) patients were given i.v. Abatacept 10 mg/kg instead of i.v. Belatacept 5 mg/kg, and 1/9 developed a Banff 1A acute rejection. The one patient given subcutaneous Abatacept did well. In a second series, 5 kidney recipients with histologically confirmed CNI nephrotoxicity were switched to subcutaneous Abatacept 125 mg weekly (15). No rejection episodes or DSA appearance were observed after a median of 9 (5–17) months. Two patients did experience reactivation of Cytomegalovirus, which is also seen in Belatacept-treated patients.

Despite all the problems and the enormous burden on the global health system, which persists to this day, the pandemic also brought forward some innovative ideas. Similar to the fruitful lessons learned from remote patient monitoring and telemedicine during the COVID pandemic, the unique experience of conversion to Abatacept during the early COVID outbreak (4) may have rekindled the interest in this agent for kidney transplantation. While the encouraging observations reported herein from a relatively large number of kidney recipients have piqued the interest for further study, the relatively short treatment interval of 3 months and lack of controls necessitates caution and the need for more evidence of safety and efficacy.

With this in mind, one center has embarked on a randomized controlled phase 2b conversion trial from Belatacept to Abatacept, entering kidney transplant recipients stable for at least 2 years on Belatacept and off all CNI drugs for at least 6 months (ClinicalTrials.gov NCT04955366). Such trials should be encouraged and perhaps multi-center collaboration can be envisioned in the near future to seek a proper role for Abatacept in the transplant immunosuppressive drug armamentarium. At a minimum, the availability of Abatacept as a backup would also be a welcomed addition during periods of Belatacept production shortages.
